# Epigenetic inheritance of telomere length in wild birds

**DOI:** 10.1371/journal.pgen.1007827

**Published:** 2019-02-14

**Authors:** Christina Bauch, Jelle J. Boonekamp, Peter Korsten, Ellis Mulder, Simon Verhulst

**Affiliations:** 1 Groningen Institute for Evolutionary Life Sciences, University of Groningen, Groningen, The Netherlands; 2 Department of Animal Behaviour, Bielefeld University, Bielefeld, Germany; The University of North Carolina at Chapel Hill, UNITED STATES

## Abstract

Telomere length (TL) predicts health and survival across taxa. Variation in TL between individuals is thought to be largely of genetic origin, but telomere inheritance is unusual, because zygotes already express a TL phenotype, the TL of the parental gametes. Offspring TL changes with paternal age in many species including humans, presumably through age-related TL changes in sperm, suggesting an epigenetic inheritance mechanism. However, present evidence is based on cross-sectional analyses, and age at reproduction is confounded with between-father variation in TL. Furthermore, the quantitative importance of epigenetic TL inheritance is unknown. Using longitudinal data of free-living jackdaws *Corvus monedula*, we show that erythrocyte TL of subsequent offspring decreases with parental age within individual fathers, but not mothers. By cross-fostering eggs, we confirmed the paternal age effect to be independent of paternal age dependent care. Epigenetic inheritance accounted for a minimum of 34% of the variance in offspring TL that was explained by paternal TL. This is a minimum estimate, because it ignores the epigenetic component in paternal TL variation and sperm TL heterogeneity within ejaculates. Our results indicate an important epigenetic component in the heritability of TL with potential consequences for offspring fitness prospects.

## Author summary

Telomeres are DNA-protein structures at chromosome ends and a short telomere length predicts reduced survival in humans, birds and other organisms. Variation in telomere length between individuals is thought to be largely of genetic origin, but telomere inheritance may be unusual because not only genes regulating telomere length are inherited, but a fertilised cell already has a telomere length (from the parental gametes). Using long-term individual-based data of jackdaw families (a small corvid species), we found that as fathers aged, they produced chicks with shorter telomeres. This suggests that telomere length inheritance has an epigenetic component. To investigate to what extent telomere length in the fertilised cell affects telomere length after birth, we compared telomere length over years within fathers with the telomere length of their consecutive offspring. This epigenetic component explained a substantial part (≥ one third) of the telomere length inheritance; whereas there was no such effect of maternal telomere length. The sex difference fits the idea that lifelong sperm formation leads to change in telomere length of the sperm cells, whereas female gametes are all formed before birth and their telomere length does not change over time.

## Introduction

Telomeres are evolutionarily conserved DNA sequence repeats, which form the ends of chromosomes together with associated proteins and contribute to genome stability [1]. Telomeres shorten due to incomplete replication during cell division, which can be accelerated by DNA and protein damaging factors and attenuated or counter-acted by maintenance processes, mainly based on telomerase activity, a telomere-elongating ribonucleoprotein [2]. On the organismal level, telomere length (TL) generally declines with age and short TL relates to ageing-associated disorders and reduced survival in humans [3,4] and other organisms [5,6]. Given this relationship of telomeres with health and lifespan it is of importance to understand how variation in TL among individuals arises, which is already present early in life [7–9].

**Fig 1:**
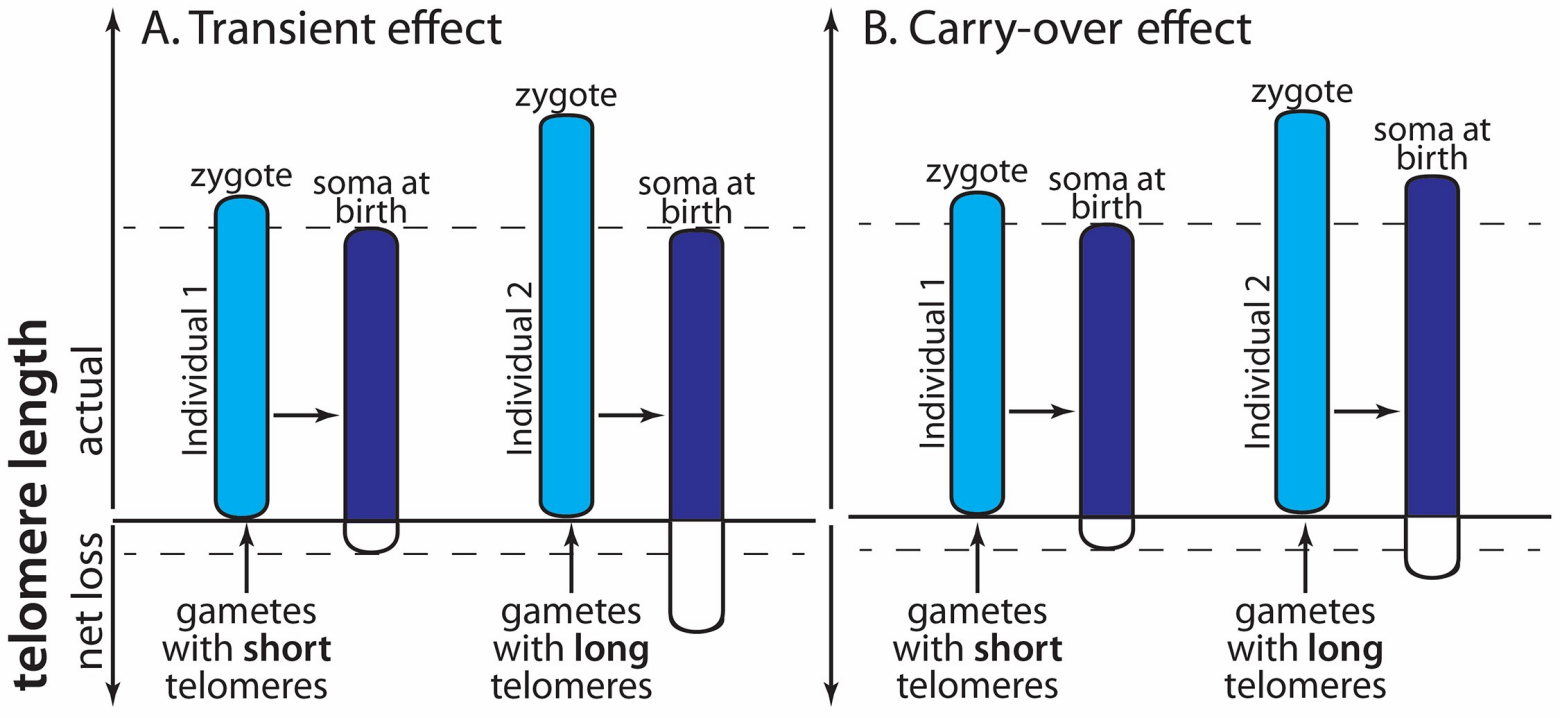
Schematic representation of inheritance of telomere length (TL). Shown is the TL of two individuals that differ in TL at conception due to a TL difference received via the gametes. Such a difference can arise through chance processes during gamete formation. Subsequently, cell division related shortening and telomerase-based restoration processes act on TL [2]. In our example this results in a net loss, but the essence is that initial differences in TL become smaller during development. Variation in genes regulating these processes contributes to the genetic inheritance of TL. If full compensation were achieved, this would result in a transient effect (A) in the sense that TL at birth is independent of variation in gamete TL. However, if variation in gamete TL persist in later life, e.g. is still present at birth independent of genes that regulate TL during development, this leads to an epigenetic carry-over effect (B).

TL has a genetic basis, but heritability estimates for TL are highly variable [10]. Compared with other traits, inheritance of TL is also unusual in that the TL phenotype is directly expressed in the zygote without any effect of its own genome. This is because the zygote’s set of chromosomes carries the telomeres of the two parental gametes. Subsequently, during development of the embryo, different telomere maintenance and restoration mechanisms, under the control of multiple genes, potentially regulate TL, but this process is poorly understood [11]. In the course of early development, such mechanisms can potentially compensate fully for gamete derived differences in TL (as suggested by e.g. [12,13]), in which case the effect of gamete TL is transient (Fig 1A). Alternatively, differences in gamete TL are carried over to later life (as suggested by e.g. [14]; Fig 1B). The latter case would imply the inheritance of parental TL, which is independent of DNA sequence variation (in vertebrates (TTAGGG)_n_ [15]), but a change in telomere sequence length (n). We interpret this as a form of epigenetic inheritance component on TL [16,17]. Note that this epigenetic inheritance mechanism differs from better known epigenetic mechanisms such as DNA methylation in that it does not affect the phenotype (TL) by modulating gene expression, but instead through direct inheritance of the phenotype itself and therefore has also been referred to as “epigenetic-like” [17].

Strongest evidence for an epigenetic mechanism of TL inheritance comes from studies that show a relationship between parental (usually paternal) age and offspring TL [18–23] with a particularly interesting example showing a cumulative effect over generations in humans [24]. In humans, where offspring TL increases with paternal age, this trend parallels a qualitatively similar change in sperm TL with age, which is generally assumed to underlie the TL increase in offspring [25]. However, studies of parental age effects in other species show mixed results and trends differ in direction between and within taxa [20,26]. More importantly, some critical uncertainties remain unresolved in any species. Firstly, studies to date are all cross-sectional [18–23], thus, comparing offspring of different parents that reproduced at different ages. Such cross-sectional trends may differ from age related changes within individual parents if, for example, individuals with long TL are more likely to reproduce at older ages, which is not unlikely given the positive correlation between human TL and reproductive lifespan [27,28].

Secondly, parental age effects on offspring TL may arise from effects of parental age on pre- and postnatal conditions prior to sampling. Because telomere attrition is highest early in life e.g. [29,30], these effects can be substantial, as illustrated by parental age effects on TL dynamics during the nestling phase in European shags *Phalacrocorax aristotelis* [31] and Alpine swifts *Apus melba* [21]. Lastly, due to their cross-sectional character, studies to date could not test whether changes in TL within parents over their lifetimes are predictive of changes in TL of the offspring in relation to parental age at conception. These points need to be resolved to establish whether the correlations between parental age and offspring TL can be attributed to epigenetic inheritance of TL, and before we can begin to understand why parental age effects on offspring TL appear to differ between and within taxa [20,26].

To investigate whether offspring TL changes with parental age at conception over the lifetime of individual parents we used our long-term, individual-based dataset of free-living jackdaws *Corvus monedula*. Telomere length was measured in nucleated erythrocytes using terminal telomere restriction fragment analysis [32] from multiple chicks of the same parents that hatched up to 9 years apart. As telomere attrition is highest early in life, we took blood samples for telomere analysis shortly after hatching, when the oldest chick in a brood was 4 days old. To test if TL was influenced by age-dependent parental care prior to sampling, we cross-fostered clutches between nests immediately after laying and tested whether foster parent age affected offspring TL. To investigate if the rate of telomere attrition within parents predicts the change in TL of the offspring they produce over consecutive years, we measured TL of the parents repeatedly over their lifetimes.

For the first time, we here show that offspring TL declines as individual fathers age and that the change in TL over time in fathers is reflected in the TL of their offspring, which explains a substantial part of the telomere resemblance between fathers and offspring and can be interpreted as an epigenetic component in the inheritance of TL. Mother offspring resemblance on the other hand was independent of maternal age and within mother variation in TL was not associated with variation in the TL of her offspring.

## Results

Descriptive data of the study population are summarised in Table 1.

**Table 1:** Descriptive statistics for our dataset of studied jackdaws.

offspring:	
n individuals	715
n nests	298
n cross-fostered (individuals/nests)	61/31
age in days (range; mean±SD)	2–4; 3.7±0.6
fathers:	
n individuals	197
n individuals ≥ 2 years	66
n individuals ≥ 2 female partners	32
age in years (range; mean±SD)	1–13; 3.5±2.0
mothers:	
n individuals	194
n individuals ≥ 2 years	62
n individuals ≥ 2 male partners	32
age in years (range; mean±SD)	1–11; 3.6±2.2

### Parental age and offspring telomere length

**Fig 2:**
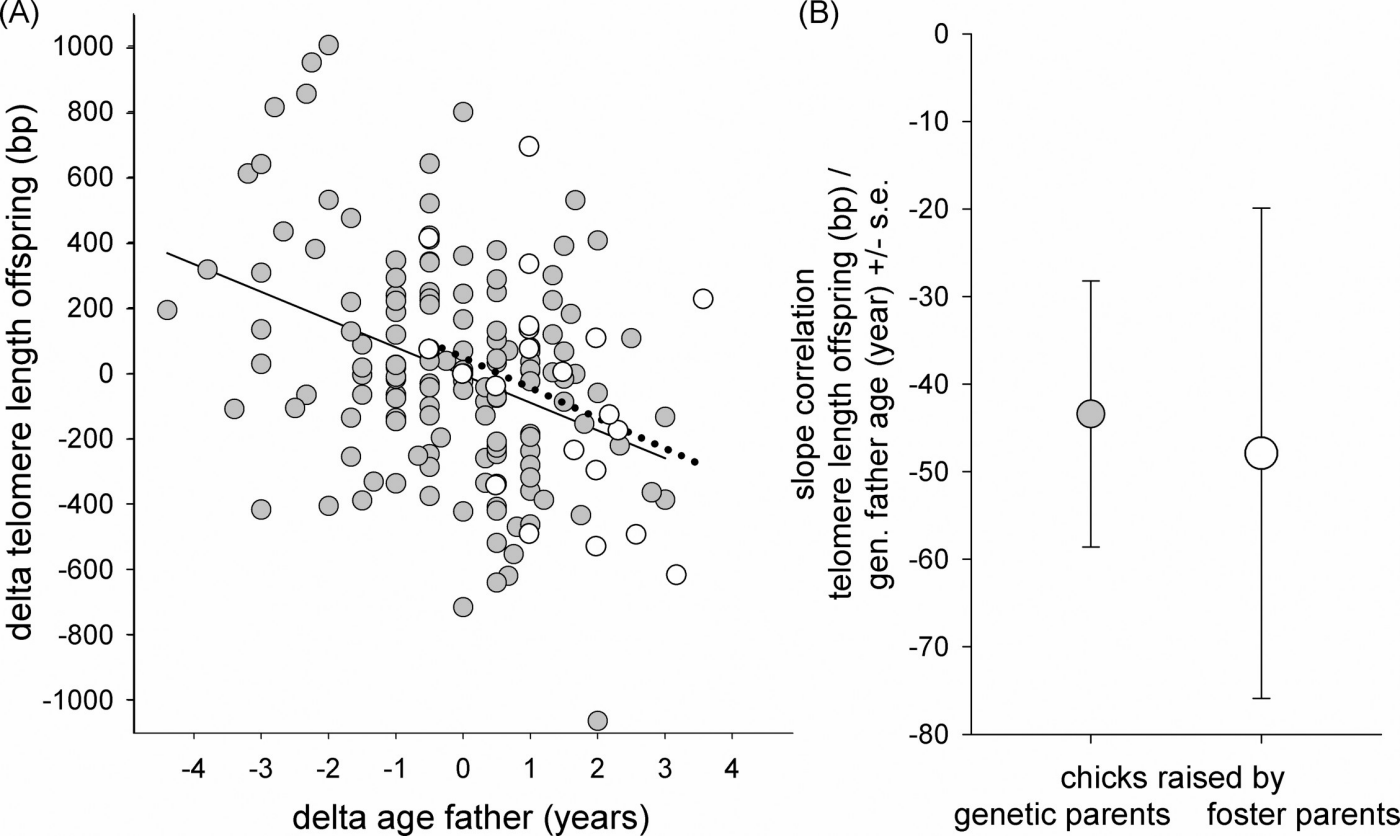
Offspring TL in relation to the age of the genetic father. (A) Delta offspring TL versus delta age of the father. Data on both axes are expressed as delta, i.e. the difference relative to the mean, for age of fathers (X-axis) or TL per brood (Y-axis) over the different sampling years. Closed symbols and solid regression line for offspring from non-cross-fostered clutches, open symbols and dotted regression line for TL of offspring from cross-fostered clutches. As cross-fostering has been performed only in recent years, open symbols are not equally distributed over the x-axis. (B) Slope values (±SE) from Table 3. Decline of early life TL of offspring produced by the same fathers over years when raised by either genetic or foster parents. Similarity of the slopes indicates that the paternal age effect on offspring TL was established prior to cross-fostering, presumably at conception.

To be able to separately evaluate between- and within-individual patterns of parental age, we used within-subject centering [33]. Instead of using age in our models, we used the mean age per individual over multiple years as one variable, and delta age, the deviation from that mean as a second variable. Thus, the coefficient of mean age estimates the parental age effect compared between individual parents, while the coefficient of delta age estimates the age effect on offspring TL within parents. As fathers aged, they produced offspring with 56±20 bp shorter TL for each additional year (variable ‘delta age father’ in Table 2A, Fig 2A), showing that offspring TL declined with paternal age at conception within individual males. This effect was not apparent when comparing offspring of different fathers reproducing at different ages (cross-sectional component of the statistical model, variable ‘mean age father’ in Table 2A). In contrast, there was no effect of maternal age on offspring TL (Table 2B), neither when compared cross-sectionally, between offspring of different mothers over age (mean age mother, Table 2B), nor within mothers as they age (delta age mother). The negative, non-significant effect of maternal age on offspring telomere length we observed (Table 2B) we attribute to the age of their mates, because pair bonds in jackdaws are maintained over many years (pers. obs.) and hence maternal and paternal age are correlated. This interpretation is confirmed by the finding that the observed maternal age estimate (delta age mother) is close to what would be expected based on the estimate found in fathers and the observed correlation of r = 0.75 (n = 298) between maternal and paternal age (i.e. 0.75 * 56 bp = 42 bp, which is very close to the estimate ± s.e. for delta mother age, which was 38±23 bp; Table 2; see also [23]). Thus, we conclude a maternal age effect on offspring TL other than through the age of the females’ mates to be unlikely. The decline in offspring TL with fathers’ age was lower than the rate of TL attrition in the fathers themselves (-56±20 versus -87±15 bp/year, respectively). Individual variation in telomere attrition slopes was negligible both between individual fathers (additional variance explained by random slopes 1%) and in their offspring produced across the fathers’ lifetimes as well (variance explained by random slopes 0.3%).

**Table 2:** Linear mixed effects model to test the effect of father (A) or mother (B) age (years) on offspring telomere length (bp). Parental age was split into two components: the mean age per parent (‘mean age father or mother’), and the deviation from that mean (‘delta age father or mother’), providing information on the effect of between parent variation or within parent variation over years, respectively. See also Fig 2A.

offspring TL	terms in model	estimate	s.e.	df	*t*	*p*
(A)	(intercept)	7228.3	132.1	667.9	54.74	<0.001
n = 715	offspring age	-31.8	29.3	590.6	-1.09	0.278
	mean age father	-26.4	19.0	189.3	-1.39	0.165
	delta age father	-55.5	20.2	80.5	-2.75	0.007
	Variance explained by random intercepts: father ID 25.3%, nest ID 11.6%, gel ID 19.7%; random slopes: delta age father 0.3%. Model fit: R^2^ = 0.578.					
(B)	(intercept)	7259.0	129.0	668.1	56.26	<0.001
n = 715	offspring age	-32.9	29.0	592.9	-1.14	0.257
	mean age mother	-31.0	17.5	193.4	-1.77	0.078
	delta age mother	-38.4	23.5	27.5	-1.64	0.113
	Variance explained by random intercepts: mother ID 31.7%, nest ID 4.4%, gel ID 18.8%.; random slopes: delta age mother: 2.6%. Model fit: R^2^ = 0.585.					

### Test for early-life paternal age effects on offspring TL

The paternal age effect on offspring TL could potentially be caused by age-dependent paternal care (e.g. age-related feeding of the incubating partner, or the chicks prior to sampling), if this affects telomere dynamics between conception and the sampling age of 4 days. We tested this hypothesis by exchanging clutches between pairs shortly after clutch completion. Our analysis is based on telomere data of 61 chicks that hatched from 31 cross-fostered clutches. In a first test, we added the age of foster father or mother to the model in Table 2A, and neither parental age significantly affected offspring TL (age foster father: 5.6±28.5, p = 0.85; age foster mother: -9.7±17.4, p = 0.58). To avoid basing a conclusion solely on a negative statistical result, in a second analysis we compared the estimate of the age of the caring father (i.e. the genetic father if not cross-fostered) on offspring TL with the estimate of the age difference between genetic and foster father (which is 0 in case of no cross-fostering or matching ages between genetic and foster father) on offspring TL. Both estimates were negative and very similar (Table 3, Fig 2B). Because the age of the caring father and the age difference between the caring father and the genetic father add up to the age of the genetic father for the cross-fostered offspring, the similarity of the estimates implies that there was no effect of age-related care between conception and sampling on offspring TL. While the estimate of the age difference did not quite reach statistical significance in a two-tailed test (p<0.09), we consider the similarity of the estimates (10% difference) the more salient result. Thus, the older the father, the shorter the TL of his offspring, independent of the age of the male that cares for the eggs and offspring up to sampling. These results show that the paternal age effect on offspring TL is explained by the age of the genetic father and that the influence of the age of the foster fathers on offspring TL at age 4 days is negligible.

**Table 3:** Linear mixed effects model to test for early-life paternal care effects associated with father age (years) on offspring telomere length (bp). Comparison of the effect of the age of the caring father (either the genetic father or the foster father when the clutch was cross-fostered) and the difference of genetic father age and foster father age (difference father ages). Note that the age of the male caring for the clutch, and the age difference between the genetic and the caring father add up to the age of the genetic father.

offspring TL	terms in model	estimate	s.e.	df	*t*	*p*
n = 715	(intercept)	7265.2	136.0	95.5	53.41	<0.001
	offspring age	-34.9	29.4	581.1	-1.19	0.236
	genetic or foster father age	-43.4	15.2	299.1	-2.85	0.005
	difference father ages	-47.9	28.0	216.3	-1.71	0.089

Variance explained by random intercepts: genetic father ID 21.5%, nest ID 13.2%, gel ID 17.8%, analysis year 5.2%.

Model fit: R^2^ = 0.578

### Parental and offspring telomere length

**Fig 3:**
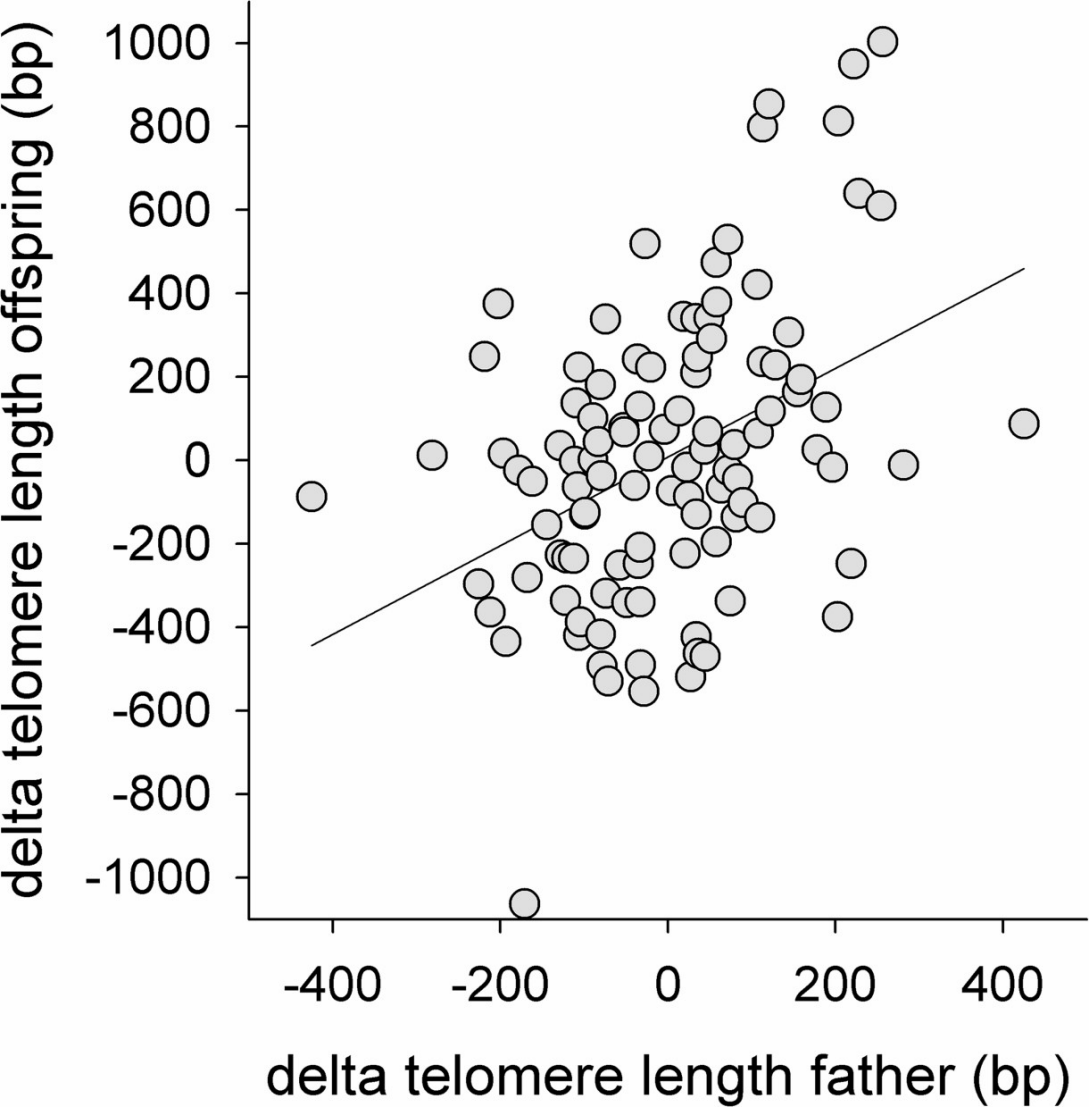
Offspring TL in relation to within individual variation in TL of their fathers at conception. Data on both axes are expressed as delta TL, the difference relative to the mean TL of fathers (X-axis) or their offspring (Y-axis) over the different sampling years. See Table 4A for the statistics.

The paternal age effect on offspring TL raises the question whether changes in paternal TL with age predict the change in early life TL of the offspring produced over the fathers’ lifetimes. We tested this by replacing the two age terms in the model in Table 2 by TL at conception (i.e. mean and delta TL) of the father in the year the offspring hatched. Fathers’ mean TL as well as delta TL were strongly and positively correlated with offspring TL (Table 4A, Fig 3). The effect of father’s mean TL on offspring TL can be attributed to additive genetic inheritance, possibly augmented by effects of a shared environment [10]. The effect of fathers’ delta TL on offspring TL cannot be attributed to a genetic effect, because delta TL refers to variation of TL within fathers over their lifetime. We therefore consider an epigenetic effect the most likely explanation for the effect of fathers’ delta TL on offspring TL. The variance in offspring TL explained by mean and delta TL of the father was 1.87 and 0.96 respectively. This indicates that 34% (0.96 / 2.83) of the variance in offspring TL that was explained by paternal TL can be attributed to the paternal-age related epigenetic effect. In agreement with our finding that maternal age did not affect offspring TL, when we performed the same analysis for mother TL, we found that maternal TL shortening (delta TL mother) was not related to the TL of her subsequent offspring, with a slope of the variable delta maternal age that was more than 90% lower than the comparable slope in males (Table 4B). However, mean maternal TL, reflecting a similarity between maternal and offspring TL per se (independent of a maternal TL change over time), based on a combination of additive genetic and age-independent maternal effects, was highly significant (Table 4B).

**Table 4:** Linear mixed effects model to test the effects of father (A) or mother (B) telomere length (bp) on their offspring telomere length (bp). Note that parental TL variation was split in two components: the mean TL over all measurements per parent (‘mean TL father or mother’), and the deviation from that mean in each of the years he/she produced offspring sampled that year (‘delta TL father or mother’). The coefficient of mean TL provides information on the effect of between parent variation, while the coefficient of delta TL provides information on variation over the years within parent. See also Fig 3.

offspring TL	terms in model	estimate	s.e.	df	*t*	*p*
(A)	(intercept)	5587.1	484.0	115.78	11.54	<0.001
n = 337	offspring age	-18.3	46.5	285.94	-0.39	0.695
	mean TL father	258.0	77.7	87.82	3.32	0.001
	delta TL father	827.2	347.3	26.47	2.38	0.025
	Variance explained by: random intercepts: father ID 6.0%, nest ID 0%, gel ID 3.3%; random slopes: delta father TL: 80.7%. Model fit: R^2^ = 0.541.					
(B)	(intercept)	4485.5	538.9	73.75	8.32	<0.001
n = 249	offspring age	-13.9	47.0	189.13	-0.30	0.767
	mean TL mother	463.9	90.8	57.97	5.11	<0.001
	delta TL mother	64.9	249.8	188.45	0.26	0.795
	Variance explained by: random intercepts: mother ID 35.7%, nest ID 0%, gel ID 26.1%; random slopes: delta mother TL: 0.2%. Model fit: R^2^ = 0.680					

## Discussion

Resemblance of TL between parents and offspring is potentially due to a dual inheritance mechanism, with on the one hand a ‘classic’ additive genetic effect and on the other hand an epigenetic effect of variation in TL in the gametes that at least in part carries through to later life (Fig 1). Suggestive evidence for an epigenetic contribution to the inheritance of TL comes from studies showing a paternal age effect on offspring TL, but available results are based on cross-sectional analyses [18–23]. Using a unique longitudinal dataset on free-living birds, and a high precision TL measurement technique (CV within individuals <3%), we show for the first time that offspring TL changes with age within individual fathers (i.e. longitudinally). We used a cross-foster experiment to test whether the paternal age effect may be due to paternal age-dependent parental care prior to offspring sampling. This showed that the paternal age effect is already present at laying. Mother age was not significantly associated with offspring TL, and the non-significant estimate of the maternal age effect matched almost exactly the expected estimate based on the observed paternal age effect in combination with the correlation between the ages of pair members. Thus, we conclude that offspring TL declined with parental age within individual fathers, but not mothers.

The parental sex dependent age effect on offspring TL is in agreement with most other studies [18–23,25,34], and is usually attributed to the different replicative history of the gametes of the two sexes. Male gametes are newly formed throughout reproductive life, while a female’s complete stock of gametes is formed before birth [35,36]. Hence TL of female gametes is less prone to changes with female age compared to TL of male gametes [37–39, but 40]. This is not to say that there is no epigenetic inheritance of TL through the female line, but only that its contribution to offspring TL does not depend on mothers’ age.

While we consider epigenetic inheritance of TL via a carry-over effect from paternal gamete TL the most parsimonious explanation of our findings, we acknowledge that we cannot yet fully exclude other mechanisms. There is some scope for females to modulate the contents of their eggs, which may affect TL dynamics [41]. Thus, it remains a possibility that females adjusted the content of their eggs in response to the age of their partner in a way that causes the paternal age effect on the TL of their offspring. However, if there were such an effect, one would perhaps also expect it to be expressed in egg volume (which varies considerably in jackdaws), but there was no evidence that females adjusted the volume of their eggs to the age of their partner (p = 0.35, n = 683 clutches, model including female identity and year as random effects). Another mechanism we cannot rule out is paternal age dependent expression of genes that control telomere dynamics of offspring. However, genetic influences on telomere dynamics are modest compared to environmental influences or heritability of TL itself [42], making it unlikely that this hypothetical mechanism explains a substantial part of the paternal age effect.

We tentatively estimated the relative contributions of additive genetic and epigenetic effects to the resemblance between males and their offspring using a statistical model in which we separated between- and within-individual variation in parental TL as predictors of offspring TL. In this model, the within-male component (‘delta TL father’, Table 4A) shows the strong epigenetic effect over the years within males on their offspring, while the between male component (‘mean TL father’) shows the putative additive genetic effect on offspring TL. When comparing the relative contributions of the two inheritance mechanisms, it appeared that 34% of the variance explained by paternal TL can be attributed to the epigenetic effect. Telomere loss within mothers (‘delta TL mother’) was unrelated to the TL of offspring produced over years (Table 4B). Estimates of the between-male effect (0.26±0.08, ‘mean TL father’, Table 4A) and the between-female effect (0.46±0.09, ‘mean TL mother’, Table 4B) together equate to a narrow sense heritability of jackdaw TL of 0.72, which is similar to results observed in humans [43] and within the range observed in other vertebrates [10] and is in line with other studies on birds estimating higher similarity between mothers and offspring [44,45]. We stress however that we measured telomere length in parental blood and not in sperm and that the estimates for the additive genetic and the epigenetic effects are tentative. Firstly, with respect to the additive genetic effect, it is of importance that shared environment effects are not controlled for in the present analysis. We note however that a more extensive analysis using multigenerational pedigree information and controlling for shared environmental effects [46] yielded a very similar estimate of the narrow sense heritability of TL in our study population (Bauch et al. in prep). Secondly, the variance in TL between males is not only of genetic origin, given that in addition there appears to be an epigenetic contribution to the between-male variance. Thus, the effect of ‘mean TL father’ (Table 4A) will to an unknown extent contribute to the epigenetic effect, as well as heterogeneity of sperm TL in ejaculates. Hence the epigenetic contribution to the resemblance between father and offspring TL will be more than the 34% we estimated based on parent-offspring regression over a single generation.

Narrow sense heritability of human TL has been estimated using monozygotic and dizygotic twins [e.g. 47], assuming that a weaker resemblance between dizygotic twins compared to monozygotic twins can be attributed to the difference in genetic relatedness. However, as monozygotic twins develop from a single zygote, and hence from a single sperm cell and oocyte, the difference in resemblance within a monozygotic versus a dizygotic twin pair may in part be due to an epigenetic effect of having developed from the same or different gametes [14]. This process would lead to an overestimation of the narrow sense heritability compared to techniques that do not depend on twins.

The direction of the paternal age effect in jackdaws (decreasing) is opposite to the direction of the paternal age effect in humans and chimpanzees (increasing) [20]. Assuming that paternal age effects in humans and chimpanzees [20] on the one hand and several bird species (including our study species) [20–23] and lab mice [34] on the other hand all reflect paternal age effects on sperm TL, this raises the question why these age effects on sperm TL are in opposite directions. Seasonality of reproduction may well play a role, with species that produce sperm for a small part of the year having less need to maintain sperm TL than species with year-round sperm production [20]. The lengthening of TL in human sperm with age has been interpreted as the result of an overshoot in telomere maintenance [25] that can be viewed as a safety margin in the maintenance process. Such a safety margin can be expected to be larger when the rate of sperm production and hence telomere attrition is higher. This may explain why chimpanzees, with a higher sperm production rate than humans, due to their promiscuous mating system, show a steeper paternal age effect on offspring TL compared to humans [48]. Information on the sign of the association between paternal age and offspring TL in strongly seasonal mammal species and / or continuously reproducing bird species would allow a test of this hypothesis.

The epigenetic inheritance of TL potentially has more general implications. Parental age at conception has previously been shown to have negative effects on offspring fitness prospects in diverse taxa, a phenomenon known as the *Lansing effect* [22,49–52]. The underlying mechanisms are likely to be diverse, but in taxa where the paternal age effect on offspring TL is negative, given that TL predicts survival in wild vertebrates [6], and TL early in life correlates strongly with TL in adulthood in jackdaws [7], offspring born to older fathers may have a shorter life expectancy due to their epigenetically inherited shorter TL. A further implication is that there may be cumulative changes in TL over multiple generations [24]. This could lead to population level changes in TL when the age structure of the population changes, as has for example been observed in birds in response to urbanisation [53]. A population level change in TL may in itself have further demographic consequences [54], providing a positive or negative feedback, depending on whether increasing paternal age has a positive or negative effect on offspring TL.

## Materials and methods

### Ethics statement

Data were collected under license of the animal experimentation committee of the University of Groningen (Dierexperimenten Commissie, DEC, license numbers: 4071, 5871, 6832A). License was awarded in accordance with the Dutch national law on animal experimentation (“Wet op de dierproeven”) and research was carried out following the guidelines of the Association for the Study of Animal Behaviour (ASAB) [55].

### Data and blood sample collection

Life-history data and blood samples originate from an individual-based long-term project on free-living jackdaws *Corvus monedula* breeding in nest boxes south of Groningen, the Netherlands (53.14° N, 6.64° E). Jackdaws produce one brood per year with mostly 4 or 5 chicks. They are philopatric breeders and socially monogamous with close to zero extra-pair paternity as shown in different populations [56,57]. Females incubate the eggs, while males feed their female partners. Chick provisioning is shared by the sexes. Each year, during the breeding season around the hatching date nest boxes were checked daily for chicks. Freshly hatched chicks were marked by clipping the tips of the toenails in specific combinations and therefore the exact ages of offspring were known. Between 2005 and 2016, 715 jackdaw chicks were blood sampled when the oldest chick(s) was (were) 4 days (note that chicks hatch asynchronously). These chicks originated from 298 nests, of 197 different fathers, whereof 66 were blood sampled repeatedly over years (max. difference of age between offspring 8 years) and 194 different mothers, whereof 62 were blood sampled repeatedly over years (max. difference of age between chicks 9 years; see Table 1 for more information). 61 chicks (that contributed telomere data) hatched from 31 cross-fostered nests, i.e. eggs were exchanged between nest boxes (selected for equal clutch sizes and laying dates (or up to one day difference), but otherwise randomly) soon after clutch completion. 54 (89%) of those chicks were fostered by a father of different age. Jackdaws in this project are marked with a unique colour ring combination and a metal ring. Parents were identified by (camera) observation during incubation and also later during chick rearing when caught for blood sampling (by puncturing the *vena brachialis*). Unringed adults were caught, ringed and assigned a minimum age of 2 years, as this is the modal recruitment age of breeders that fledged in our study colony. All jackdaws were of known sex (molecular sexing [58]).

### Telomere analysis

Blood samples were first stored in 2% EDTA buffer at 4–7°C and within 3 weeks snap frozen in a 40% glycerol buffer for permanent storage at -80°C. Terminally located telomere lengths were measured in DNA from erythrocytes performing telomere restriction fragment analysis under non-denaturing conditions [29]. In brief, we removed the glycerol buffer, washed the cells and isolated DNA from 5 μl of erythrocytes using CHEF Genomic DNA Plug kit (Bio-Rad, Hercules, CA, USA). Cells in the agarose plugs were digested overnight with Proteinase K at 50°C. Half of a plug per sample was restricted simultaneously with *Hind*III (60 U), *Hinf*I (30 U) and *Msp*I (60 U) for ~18 h in NEB2 buffer (New England Biolabs Inc., Beverly, MA, USA). The restricted DNA was then separated by pulsed-field gel electrophoresis in a 0.8% agarose gel (Pulsed Field Certified Agarose, Bio-Rad) at 14°C for 24h, 3V/cm, initial switch time 0.5 s, final switch time 7.0 s. For size calibration, we added ^32^P-labelled size ladders (1kb DNA ladder, New England Biolabs Inc., Ipswich, MA, USA; DNA Molecular Weight Marker XV, Roche Diagnostics, Basel, Switzerland). Gels were dried (gel dryer, Bio-Rad, model 538) at room temperature and hybridized overnight at 37°C with a ^32^P-endlabelled oligonucleotide (5’-CCCTAA-3’)_4_ that binds to the single-strand overhang of telomeres of non-denatured DNA. Subsequently, unbound oligonucleotides were removed by washing the gel for 30 min at 37°C with 0.25x saline-sodium citrate buffer. The radioactive signal of the sample specific TL distribution was detected by a phosphor screen (MS, Perkin-Elmer Inc., Waltham, MA, USA), exposed overnight, and visualized using a phosphor imager (Cyclone Storage Phosphor System, Perkin-Elmer Inc.). We calculated average TL using ImageJ (v. 1.38x) as described by Salomons *et al*. [29]. In short, for each sample the limit at the side of the short telomeres of the distribution was lane-specifically set at the point of the lowest signal (i.e. background intensity). The limit on the side of the long telomeres of the distribution was set lane-specifically where the signal dropped below Y, where Y is the sum of the background intensity plus 10% of the difference between peak intensity and background intensity. We used the individual mean of the TL distribution for further analyses. Samples were run on 92 gels. Repeated samples of adults were run on the same gel, chicks were spread over different gels. The coefficient of variation of one control sample of a 30-day old jackdaw chick run on 26 gels was 6% and of one control sample of a goose, with a TL distribution within a similar range, run on 31 other gels was 7%. The within-individual coefficient of variation for samples run on the same gel was <3% [7] and the within-individual repeatability of TL was estimated to be 97% [59].

### Statistical analyses

The relationships between parental age or parental TL and early-life TL of offspring were investigated in a linear mixed effects model framework using a restricted maximum-likelihood method (testing specific predictions). To be able to separately evaluate between- and within-individual patterns of parental age or parental TL, we used within-subject centering [33]. Thus, instead of father age, mother age or father TL, mother TL per se we introduced the mean value per individual over (if available) multiple years and delta age or delta TL, the deviation from that mean, respectively. To account for (genetic and potential other) similarities in TL between offspring of the same father or mother, we included father ID or mother ID as random effect in the model. As the dataset contains also siblings raised in the same nest, we additionally added a random effect of nest ID as a nested term in father ID or mother ID to the models investigating paternal or maternal age effects on TL, respectively. The age of chicks at sampling differed slightly (2–4 days) and as TL shortens with age [7], we included their age (in days) as a covariate. Offspring sex was never significant and was therefore excluded from the final models. We added gel ID as random effect. Analyses were performed separately for fathers and mothers as their ages are correlated.

The cross-foster experiment was designed to test for potential effects of parental age on early-life telomere attrition between egg laying and sampling (age 2–4 days). First, we modified the linear mixed effect model with offspring TL as dependent variable testing for paternal age effects (see above) by adding the age of the foster father or mother as covariate. Second, in a linear mixed model with offspring TL as dependent variable, we included both the age of the father caring for the clutch after cross-fostering and the age difference between the genetic father and foster father as covariates (age genetic father-age foster father). When the paternal age effect is independent of age-dependent effects between conception and sampling, we predict the coefficients of the caring father’s age and the age difference between genetic father and foster father to be indistinguishable. This is so because the age of the male caring for the clutch, and the age difference between the genetic and the caring father add up to the age of the genetic father. In contrast, when the paternal age effect is entirely due to age-dependent paternal effects after laying, the coefficient will be the same, but opposite in sign. In case of a mixture of the two effects, the coefficient will be intermediate. In this analysis we used all offspring, i.e. also those that were not cross-fostered, and further included genetic father ID, nest ID, gel ID and year of telomere analysis as random effects, and offspring age at sampling as covariate.

Statistics were performed using packages lme4 [60], lmerTest [61], MuMIn [62] in R (version 3.3.3) [63]. In the results mean ± standard error is given unless stated otherwise.

## Supporting information

S1 Dataset File containing the data underlying the findings. (XLSX)
